# The safety paradox in ethics training: a case study on safety dynamics within a military ethics train-the-trainer course

**DOI:** 10.1007/s11019-018-9847-9

**Published:** 2018-07-10

**Authors:** Eva van Baarle, Ineke van de Braak, Desiree Verweij, Guy Widdershoven, Bert Molewijk

**Affiliations:** 1 Military Ethics and Philosophy, Netherlands Defense Academy, Breda, The Netherlands; 2 Learning Network NEON, VU University Medical Centre (VUmc), EMGO+, Amsterdam, The Netherlands; 3 4D Organization Development and Education, Valkenburg aan de Geul, The Netherlands; 4 Philosophy and Ethics, Netherlands Defense Academy, Breda, The Netherlands; 5 Centre for International Conflict Analysis and Management (CICAM), Radboud University Nijmegen, Nijmegen, The Netherlands; 6 Medical Ethics and Philosophy, Department of Medical Humanities, VU University Medical Centre (VUmc), EMGO+, Amsterdam, The Netherlands; 7 Clinical Ethics, Department of Medical Humanities, VU University Medical Centre (VUmc), EMGO+, Amsterdam, The Netherlands; 8 Clinical Ethics, Centre for Medical Ethics, University of Oslo (UIO), Oslo, Norway

**Keywords:** Safety, Ethics training course, Group dynamics, Military ethics

## Abstract

There is considerable support for the idea that an atmosphere of safety can foster learning in groups, especially during ethics training courses. However, the question how safety dynamics works during ethics courses is still understudied. This article aims to investigate safety dynamics by examining a critical incident during a military ethics train-the trainer course during which safety was threatened. We examine this incident by means of a four-factor analysis model from the field of Theme-Centered Interaction (TCI). We show that during ethics training courses a safety paradox can occur, involving a tension between honesty and openness to other perspectives and values. Finally, we discuss how trainers can foster safety during ethics training.

## Introduction

Research on ethics training courses shows that an atmosphere of safety within groups is important during ethics training courses, particularly during those courses based on a dialogical, reflective and interactive approach in which participants practice with ethical reflection and deliberation themselves (Smith and Berg 1987; Knapp and Sturm 2002; Tucker et al. 2007; Molewijk et al. 2008; Abma et al. 2009; Wortel and Bosch 2011; Stolper et al. 2012; van der Dam 2012; Solum et al. 2016). However, the question of how a safe atmosphere within groups can be fostered during dialogical ethics training remains understudied (Boers 2003; Weidema et al. 2011; Edmondson and Lei 2014). Although safety is crucial for learning, it is also precarious, and can easily be threatened.

Safety within a group can be defined as the feeling of a student that he or she can contribute to a dialogue or ask a critical question without fear or negative consequences, for instance to be embarrassed. These negative consequences may have an influence on self-image, social status and future career (cf. Kahn 1990). Amy Edmondson defined this as psychological safety, in groups and teams, as a shared belief that a team is safe for taking interpersonal risks. It encompasses a sense of being valued and comfortable in a specific setting (Edmondson 1999, 2004).

The aim of this article is to better understand dynamic aspects of safety within ethics training courses, specifically when safety is endangered. By means of a four-factor analysis model from the field of Theme-Centered Interaction (TCI) (Cohn 1976; van de Braak 2011) we reflect on a specific instance in which honesty by a participant resulted in conflicting or colliding views which challenged safety and mutual openness between participants. We explore how safety was threatened in this situation and what would be needed to foster it. We offer practical recommendations on how this challenge to safety can be met.

Ethics training, in a variety of contexts and organizations, can be characterized as a form of learner-centered education that focuses on educating self-critical thinkers (Hansen and Stephens 2000). This type of training includes, among other things, the processes of recognizing personal values and the values of others and assessing and judging moral dilemmas. Such a setting requires participants, who are willing to contribute to dialogues, appreciate mutual differences and are curious to learn from each other, and to allow possibly conflicting, views and personal values (Foldy 2004; Foldy et al. 2009; Weidema et al. 2011; Sims 2004; Sims and Felton Jr. 2006). To achieve this, an atmosphere of safety is required.

It has been argued that safety within groups has a dynamic character, coming and going (Sims 2004; Edmondson and Lei 2014). ‘It seems reasonable to assert the likelihood of an asymmetry, in which safety takes time to build, through familiarity and positive responses to displays of vulnerability and other inter-personally risky actions, but can be destroyed in an instant through a negative response to an act of vulnerability’ (Edmondson and Lei 2014, p. 38). It is perhaps particularly in those situations when safety is threatened or eroded that one becomes aware of the importance of safety during ethics training courses. The dynamic process of safety should be taken into account when trying to foster an atmosphere of safety during ethics education. For instance, by acknowledging and taking responsibility for less constructive behavior and by talking about it in the group, which ‘can make the inevitable human foibles much less destructive and offer potent teachable moments’ (Sims 2004, p. 206).

In the following, we first provide a description of the study context, methods, data collection and data analysis and subsequently introduce the four-factor analysis model. We then explore the dynamic nature of safety by analyzing a critical incident in which safety was threatened according to the participants of a military ethics train-the-trainer course. We conclude this article with a discussion of our findings, and practical implications for ethics trainers regarding safety dynamics.

## Methods

### Study context

The ethics training central to this case study is a military ethics train-the-trainer course. The objective of this course is to prepare the participants to give ethics training courses to military personnel, within the Netherlands defense organization, while at the same time fostering their own moral competence (Karssing 2000; Sherblom 2012; Wortel and Bosch 2011). The course includes topics such as different ethics theories (utilitarianism, deontology and virtue ethics), law and ethics and dilemma training or moral case deliberation (van Baarle et al. 2015). There is a strong emphasis on creating a joint dialogue within the training in order to foster personal development, on gaining (self-)awareness and being able to identify personal moral values and the moral values of others.

The military ethics train-the-trainer course is a 9-day course spread out over a 6 weeks period, consisting of three three-day units. It is an in-company training, the two trainers and participants of each group work within the Netherlands armed forces (i.e. either in the Royal Netherlands Army, the Royal Netherlands Navy, the Royal Netherlands Air Force or the Royal Netherlands Marechaussee).

Each group consists of 11–16 participants, which can be considered a relatively small group size. Before the start of the training, individual intake interviews are held with all participants. Participants are asked if they are in a hierarchical relation with other participants. If this is the case, trainers will discuss with the participant how to deal with this. Trainers also discuss this issue during the training. Participants are invited to have an open attitude during the training and to take on a vulnerable position and to put forward personal moral dilemmas in order to subsequently reflect on those dilemmas. This creates a learning environment which offers an opportunity to link theory, one’s own actions in the group, day-to-day practice and reflection on all these elements. During the course, the trainers work with Theme Centered Interaction (TCI) as a didactical method (Cohn 1976; Jaques and Salmon 2007; Stollberg 2008; van de Braak 2011).

### Selection of the critical incident

A qualitative approach, based on the Critical Incident Technique (CIT) is used as an exploratory and investigative tool to better understand how an atmosphere of safety works and how it can be fostered during ethics training (Chell 1998; Woolsey 1986; Erlandson et al. 1993). The selection of critical incidents was based on a previous study based on semi-structured in-depth interviews with participants of the train-the-trainer course with regard to the effects of this train-the-trainer ethics course (van Baarle et al. 2017). Participants mentioned the topic of safety in the group, even though the interviewers did not explicitly address this topic. Several specific moments during the course were referred to. It appeared that 10 out of 11 interviewees, who were all participants of one particular group, perceived a decrease of safety in the second block of the training (van Baarle et al. 2017). The interviewees all referred to one particular situation which caused this change. They mentioned it either in the context of safety in the group or as a situation that they found challenging.

### Data collection and analysis

**Fig. 1:**
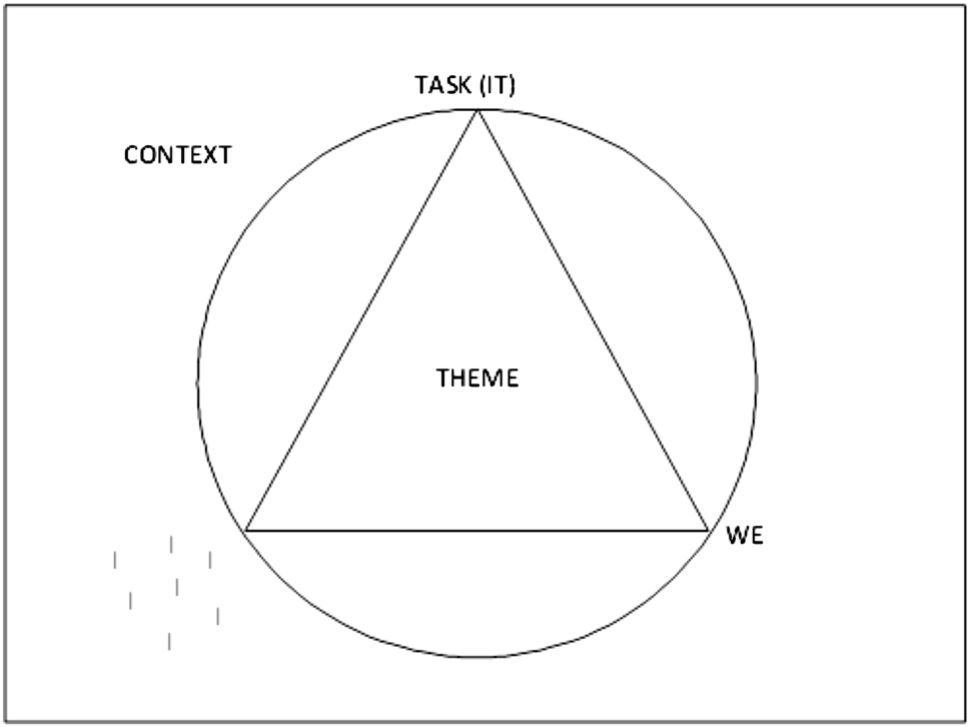
A four-factor model from Theme Centered Interaction (TCI)

The first author, who was also one of the trainers during this course, drafted a thick description (Ponterotto 2006) of this critical situation, based on notes taken during the training. To analyze the critical incident, the four-factor analysis model from TCI was used. As mentioned above, TCI is a didactical method in professional training and organizational development (Cohn 1976; van de Braak 2011; Stollberg 2008). It can also serve as a tool for analyzing processes during the training. As every training situation is singular, no general recommendations can be given for dealing with situations in which conflicts arise. Yet, the TCI’s four-factor model can serve as a framework within which factors can be analyzed that play a part in interaction processes (Fig. 1). Systematically reflecting on the four factors can provide insight into which element or elements need further attention in order to foster safety within a particular group.

**Fig. 2:**
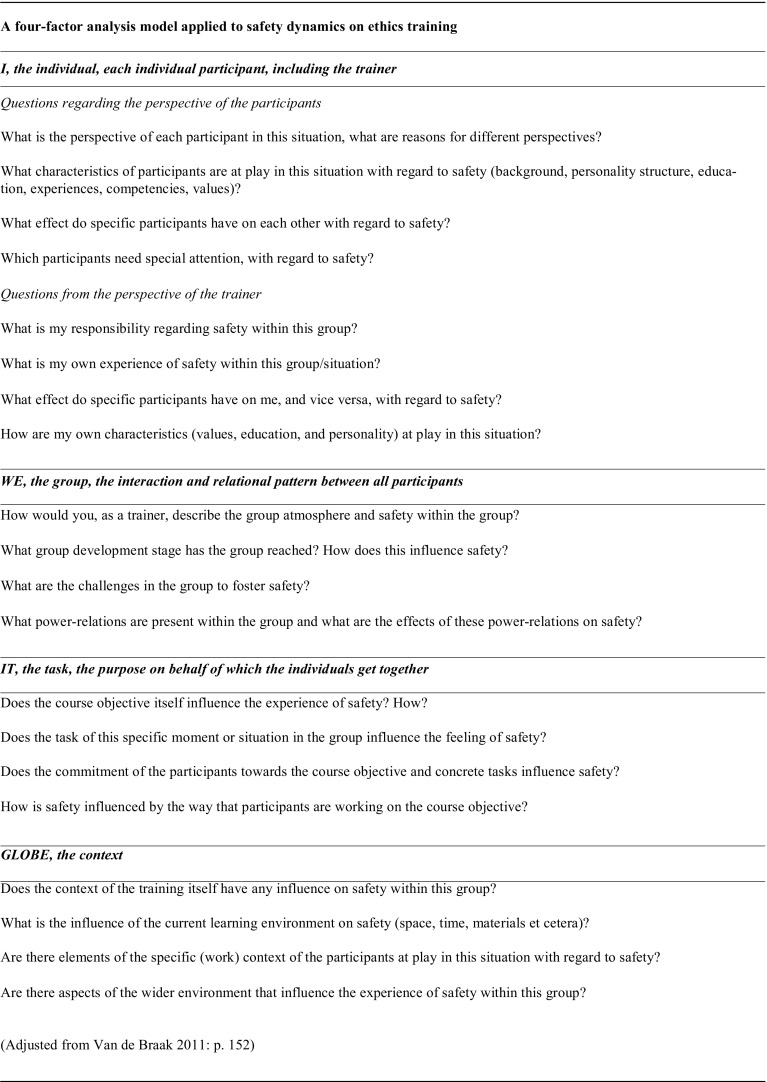
A four-factor analysis model applied to safety dynamics in ethics training

The model distinguishes four factors relevant in interaction processes. First, the individual, each single participant with his or her interests and needs, including the leader, in this case the trainers (I). Second, the group, the interaction and relational pattern between all participants (WE). Third, the task, the purpose on behalf of which the individuals get together (IT). And fourth, the context, the environment, conditions, constraints and circumstances in which cooperation takes place (GLOBE). The context includes elements such as the size of the group, the room or the arrangement of the furniture but also externally driven factors like organizational, political or social backgrounds (Cohn 1976; Jaques and Salmon 2007; Langmaack 2004; Spielmann 2009; van de Braak 2011) (Fig. 2).

### Research ethics

The interviews were recorded and the transcriptions were sent to the interviewees for a member check. It was made explicit to the participants that the data from the interviews would be processed anonymously and used in a scientific article. When referring to the interviewees in the thick description, pseudonyms are used for reasons of anonymity.

## Results

In this section we describe a critical incident in which safety was threatened according to the participants of a military ethics train-the-trainer course. In doing so, we apply the adjusted four-factor analysis to this incident in order to give a more structured and nuanced description of the critical incident.

### Critical incident

The incident took place during the first session in the second block of the train-the-trainer course on military ethics. This session is themed around the question ‘how do I act when dealing with moral issues?’ One of the participants shared his personal experience:

Ed: ‘I sometimes have a habit of ‘levelling with others’, which makes me put aside my own values, but I wonder whether this is what I really want? Daring to act on the basis of my own moral terms is also important. I want to focus on that during this training. For example, when I had just returned from a tough mission to Afghanistan for which I’d only had two weeks of preparation time, one of my colleagues started complaining that he was also going to be deployed (going on a mission) to Afghanistan. He was complaining whilst knowing my story. Knowing what a tough mission I had in Afghanistan. He was saying how he wasn’t given enough time to prepare for his deployment. While his posting meant he was up for a more or less ‘Club Med experience’ though (a relatively safe mission, without leaving the military compound or staying at the headquarters). Above that, he didn’t have a wife and children. In my eyes, he wasn’t a real military man. And I simply don’t want to work together with someone like that. He was a real good-for-nothing. People like that don’t belong in the organization’. Participants moved restlessly in their seats, not knowing how to react. One participant asked how this colleague was functioning. Ed stated: ‘He functioned okay but it didn’t take much energy to influence the director in the right direction: to get rid of this colleague … And indeed, this colleague ended up leaving. I’m outspoken when it comes to those types of time wasters. It gave me a good feeling and made me feel proud. I know I have a bit of a dark side, perhaps a bit extreme edge. But I want to be true to myself. I think it’s a good thing, to be your own judge’.

After a long silence a few people started to ask questions: *‘*Why did this make you feel proud?’ Ed: ‘I stood up for my group of ‘real’ military personnel, I have made sure that this rotten apple was now gone.’ One of the participants remarked that he could also imagine being in that situation. However, most of the other participants who spoke up seemed to openly disapprove of the case contributor’s behavior, shaking their heads. A participant stated to another participant: *‘*Isn’t this exactly why we have procedures for this from the Human Resources department?’

At this moment the two trainers doubted whether or not to continue with the next session on the program. On the one hand, there was a guest lecturer who was already waiting outside the classroom to start with the next session. On the other hand, the group was still in the middle of this discussion and there were still a number of participants who also wanted to reflect on the initial question of how to act when dealing with moral issues. One of the trainers stated: ‘While this example clearly raises a lot of questions, we will come back to this example in depth later on during the course, for instance with regard to the blurring of moral standards. For now, we will have a short break of 10 min and continue with the next session by our guest lecturer …’.

By applying the four-factor model of TCI to this critical incident, we explore how safety was threatened in this situation and how it could have been fostered. Afterwards, we will discuss a number of practical implications for trainers when aiming for safety during ethics courses.

### A four-factor analysis

#### Individual characteristics (I)

Looking at the first factor of the four-factor analysis of the critical incident, we examine how the perspectives of the participants as well as the trainers with regard to the critical incident influenced safety dynamics during the training. During the interviews, the majority of the participants of the training explicitly indicated that the incident described above negatively influenced safety during the training: They considered it inappropriate to talk about, and behave towards a colleague the way Ed did, but did not experience room to express this view. For them, values such as a respectful, honest and humane approach to colleagues appear to have been at stake in Ed’s example.

Anna: ‘I remember thinking, he has an entirely different outlook on life and I realized that I didn’t really feel comfortable sharing things with him. At that moment I thought, he is really extreme and I think he has become that way because of his experiences.’

Caroll: ‘I was in a hierarchical situation with Ed, because I depended on him for my evaluation, I wasn’t going to tell him what I thought of his actions during the course.’

Paul: ‘I think it was this intense because it [the contributed example, clarification by authors] really touched people, it touched on their core values.’

There were also participants who stated the opposite: that the case contributor was very honest to share a personal experience and that the reactions by other participants created unsafety.

Leo: ‘(…) if you just hear that without any kind of context, it’s absolutely not done, but once you hear the reasoning behind it I feel more understanding, I think people are often too quick to judge. He opened up, because that’s what we said, you have to be open and then when he is, boom, he is heavily criticized. I don’t think that is fair, you should also make an effort to understand where he’s coming from.’

Eventually, the reactions of the participants also influenced how the case contributor himself experienced safety in the group. He was criticized and felt that the other participants were unable to be open towards his perspective. He was left with the feeling that the other participants were not open to his perspective. As a consequence, Ed asked himself how safe the course environment was.

Ed: ‘During the course, I noticed that certain other participants didn’t appreciate my way of handling things. (…) I didn’t expect them [the group, note by authors] to form an opinion of me straight away, that this would still influence their perception of me days later. (…) I feel this undermined my position within the group, and it made me question how safe this environment actually was. I shared my experiences and thoughts, which may have seemed extreme to some group members, and as a result they didn’t empathize with me.’

What were the perspectives of the trainers at this moment? Based on the notes the trainers took during the training, they seemed shocked and surprised by the behavior of the contributor.

Reaction by trainer I:

The words ‘extreme edge’ sent a shiver down my spine and immediately put me on my guard. Right there and then, I decided to be extra cautious with this participant for the rest of the training.

Reaction by trainer II:

I am shocked by this example, it came out of nowhere and I wonder whether I know the colleague who was forced out.

The trainers first tried to gauge the participant’s reactions; they perceived this as a situation that should be dealt with adequately. On the other hand, there was a quest lecturer waiting to start with the next session. Values held by the trainers that were at stake in this situation are responsibility towards the participants and their professionalism, as for the trainers, this seemed as a situation which was directly related to the objective of the course (fostering moral competence). The trainers didn’t have (or took) the time to analyze this situation immediately.

#### Group dynamics (WE)

When focusing on the WE, the influence of various group dynamic elements such as the group atmosphere, challenges in the group and the development stage the group has reached, are examined. Understanding which phase of group development the group has reached can inform our analysis with regard to expectations of group members (Tuckman 1965; Weisfelt and van Andel 2005; Remmerswaal 2013; Rubner and Rubner 2016). A new group may seem very safe because nobody disagrees and conflicts do not seem to arise, whereas in fact participants may be keeping their distance and do not experience enough safety to actually reflect on moral issues and personal experiences. Most group development phase models indicate that this behavior is followed by a phase of struggle and conflict in which participants attempt to find out to what extent they can be themselves in the group. After this ‘crisis phase’ comes a phase of increased and more in-depth trust, as well as a greater willingness to work together closely, to open up and to learn. Not all groups go through every group phase as intensively, nor is this always a linear process (Remmerswaal 2013).

The critical incident took place on the first day of the second block of the training course. There was approximately 1 week in between the first and the second block of the course. The group appeared to be in the formation and orientation phase, in which most participants were still undecided between approaching one another and acting reservedly. While most participants were still hesitant to share personal experiences, the critical incident shows that the case contributor did share a personal experience in the group. This disclosure made some participants feel unsafe, while at the same time these reactions also influenced the perceived safety of the case contributor. In terms of development stages, this seemed to lead the group to a crisis phase. In crisis phases, there is a risk of participants pulling out, leading to a decrease of safety (Rubner and Rubner 2016).

In the situation described above, a risk was that the group could be split into three parts: two kinds of ‘moral crusaders’, those who condemned the reaction by the case contributor, and those who approved of his reaction and were proud of the reported behavior, and third, ‘the bystander’s: the participants who found it hard to reach a judgement at all (Rubner and Rubner 2016). If not handed adequately, such a situation might evoke a parallel process, in which the participants who seemed to have believes that were not accepted by the majority of the group, were subsequently ‘cast out’. The reaction of the case contributor seems to head in this direction, as he stated that the reactions of the other participants to his example undermined his position in the group.

#### The course objective (IT)

Turning to the course objective, the question is how the critical incident is related to the aim of fostering moral competence (i.e. the course objective of this train-the-trainer course on military ethics).

Within the course moral competence is defined as becoming aware of one’s own personal values and the values of others; the ability to recognize the moral dimension of a situation and identify which values are at stake or are at risk of violation; the ability to adequately judge a moral question or dilemma; the ability to communicate this judgment; the willingness and ability to act in accordance with this judgment in a morally responsible manner and the willingness and ability to be accountable to yourself and to others.

In order to achieve the course objective, honesty is necessary. Yet, honesty can be at odds with being open towards each other. The example brought up by the case contributor shows that he was honest about his values, but not open towards other possible values. His honest expression of his views furthermore made it difficult for other participants to be open; they regarded the way in which the case contributor expressed his views as offensive, which made it impossible for them to be open to the views of the case contributor. Thus, views collided, which negatively influenced the experience of safety of all participants.

A confrontation between values of participants seemed to be at stake. For the case contributor, his main focus seemed to be on working with fully capable members of the military, and this colleague, in his eyes was not a ‘real’ military man. At the other end of the spectrum, for some participants, values such as the respectful and fair treatment of colleagues seemed to be at stake. This led to a tension between perspectives and different sets of values voiced by the contributing participant on the one side and a number of participants on the other side. Being confronted with a diversity in values and opinions, and eventually with a clash of values in a group, is related to the core objective of the course: to foster moral competence in dealing with different moral perspectives.

The critical incident shows that differences with regard to core values participants hold can evoke strong negative feelings and can result in feelings of insecurity or a perceived lack of safety. Working on the course objective asks from participants to be open to perspectives of other participants in order to be able to fully examine and understand the underlying values at stake in a particular situation.

#### The context (GLOBE)

A number of elements in the critical incident are directly related to the context in which the training took place. We distinguish between on the one hand the influence of the learning environment in which the training took place, cultural characteristics such as formal and informal rules that directly originate from the military context, and on the other hand the fact that this was an in-company training.

In the context of the training itself, in the first block of the course, safety had been addressed. During the second day of the course, the topic of safety was brought up by one of the participants. She asked how safety would be dealt with in this group. Two participants stated that as trainers in their own courses they simply state that ‘the setting is a safe learning environment’. Since the training is a train-the-trainer course, participants were asked to reflect on their previous experiences with safe and unsafe learning environments as participants and as trainers. Eventually participants agreed that in this group they would be responsible themselves to identify boundaries of safety. Some participants stated that safety is also a feeling which they expected would ‘grow’ over time during this course.

We might also consider the wider context and its influence on the experience of safety. In a military context, rules are important, codes of conduct, mandates, rules of engagement, the law of armed conflict, human rights, are all examples of this. Not obeying these rules can have serious consequences for others, as well as for soldiers themselves. The participants are well aware of these rules and this is the precise reason why the critical incident is interesting. In stating that it felt good ‘to be your own judge’, the contributing participant did not adhere to the formal rules. The Netherlands ministry of defense code of conduct refers, among other things, to the duty not to bully, discriminate or sexually harass colleagues. This participant did not choose the easy way out by complying with political correctness and avoiding the risk of being challenged by other participants (Hansen and Stephens 2000).

The example given by the case contributor is less uncommon than one might think. In the context of the military academy, this phenomenon is known as ‘internal clean-up’ [*interne sanering*]. It is based on the idea that some military personnel are unfit for service and do not belong at the academy, let alone in a military operation: ‘Those who are militarily proficient and, therefore, good comrades are lauded, while those who have failed to contribute to collective goals are ridiculed, defaced, and ultimately, excluded’ (King 2006, p. 509). In order to also make the discussion of these kinds of informal rules possible, it is important that a sufficient level of safety is maintained to enable the group to look into these situations and to not instantly label certain contributions as extreme or pathological.

Participants and trainers all work in the same organization. As a consequence, there might be formal hierarchical work relations between participants in the course. In the critical incident one of the participants appeared to be dependent on the case contributor for her evaluation. She felt that she could not tell him what she thought of his actions during the course. The trainers were unaware of this hierarchical relation. Apart from this example, participants may foresee that they will come into contact with one another again in the future. This can act as a brake on the willingness to share experiences and to openly respond to each other during the course.

## Discussion

How do safety dynamics work during ethics training? How can safety be threatened and what can be done to foster an atmosphere of safety? In order to explore these questions, we analyzed a critical incident in which safety was under pressure during a military ethics training course, according to both the trainers and the participants of the course. By using the four-factor model we analyzed safety dynamics in a broad sense, taking into account the perspectives of the participants and the trainers, the group process, the relation with the course objective and the specific military context.

We consider two issues for the discussion. First, we go into the safety paradox, which involves a tension between honesty and openness. Second, we discuss how to deal with situations where safety is threatened during ethics training courses.

### The safety paradox

Existing research acknowledges the importance of safety in dialogical, reflective and interactive approaches to ethics training (Knapp and Sturm 2002; Abma et al. 2009; Wortel and Bosch 2011; Stolper et al. 2012; van der Dam 2012). The experience of an atmosphere in which participants feel free to share opinions and feelings, even if these contradict the point of view of the majority of the participants is considered to be valuable.

Safety can be regarded as a precondition for dialogue and reflection as it creates room for a more in-depth approach to the majority viewpoint and room to rethink this viewpoint and to appreciate the views held by others. Reflecting on different insights and values may lead to new and broader insights. In light of the course objective, dealing constructively with disagreement by using a reflective and dialogical approach to the differences and disagreements (e.g. asking questions to better understand the view of the other instead of stating and arguing for one’s own position’), again may also have the result that it contributes to experiencing more safety (Widdershoven and Molewijk 2010). As such, differences of opinions are valuable for ethics training courses. Homogeneity of opinions may initially create safety within groups (Dixon 1994), but at the same time homogeneity may also create blind spots and ‘group think’ (Argyris 1990).

The critical incident described above supports the idea that safety can indeed ‘be destroyed in an instant through a negative response to an act of vulnerability’ (Edmondson and Lei 2014, p. 38). Our results show that safety dynamics can be paradoxical: The course objective invites participants to engage in dialogue and reflection and to be vulnerable and honest about one’s own considerations. Yet, the case shows that honesty is not only a requirement for, but can also be a threat to safety. Being honest implies expressing different perspectives and values. However, honesty can result in a clash of values and make it more difficult for participants to be open towards other perspectives and underlying values. As a consequence safety may decline and be threatened. A group which is able to deal productively with this safety paradox can develop and come to a group phase of increased and more in-depth trust, as well as a greater willingness to work together closely, to open up and to learn (Remmerswaal 2013).

In the heat of the moment, it seems that neither the trainers, nor the participants, clearly identified the situation in the group as an example of a clash of values. Instead of enabling an open investigation of different values, the presentation of the case and the reaction of the participants resulted in a confrontation of viewpoints. Providing space for feelings of vulnerability, inequality or even reluctance (Weidema et al. 2011) is not self-evident; rather it demands time, courage and moral competence. The ability to identify a situation on the spot as an example of a clash of values and the willingness to understand the perspective of participants with different values seems to presuppose that participants are able to reflect on their own opinions, to examine contradicting opinions and react to each other with curiosity rather than debate and defensive behavior.

When participants experience a lack of safety in the group, the trainer can be left with the feeling that all sorts of things have gone wrong; that they have not performed well as a trainer or that perhaps it was ‘simply’ a difficult participant or a difficult group. A different approach would be to view this setting as active, both in terms of interaction and group dynamic, with the group development process being in full swing and the trainer having the responsibility and possibility to support this process. This requires knowledge and skills in interaction and group dynamics. As such, safety dynamics, including situations which may seem threatening to the experience of safety, can be viewed as an ongoing process and as an opportunity to work on the objective of ethics training courses rather than as a problem to achieve this. In the following section we will examine how safety might be fostered in the here-and-now.

### Dealing with situations where safety is threatened

Situations during an ethics training in which safety is experienced as threatened by a number of participants can be challenging and disturbing. Sometimes trainers might be surprised or overwhelmed by such situations; they have a program to follow and limited time to reflect on situations in the here-and-now. More importantly, they may lack a clear strategy on how to tackle a situation in which safety is threatened.

While there are no guarantees in terms of results or success, Smith and Berg argue that the key may be not to learn how to avoid these situations but rather to learn how to progress and avoid remaining stuck in these situations (Smith and Berg 1987). Since fostering moral competence is the main objective of many ethics training courses, situations such as described above might provide an opportunity to exercise moral competence at that very moment. The critical incident may be viewed as a concrete example of a situation where different perspectives and values collide. As such, this challenging situation can be seen as desirable. It may provide an opportunity for learning in the here-and-now (van Staveren 2007; Schruijer 2016).

Learning in the here-and-now attempts to integrate interaction, actual behavior by participants and reflection on underlying values. How can this be achieved in practice? How to use a situation as an opportunity to deal productively with the situation and work on the objective of the course at the same time?

Learning from situations where safety is threatened is well known in the tradition of the Socratic dialogue, a dialogical method often used during ethics training courses. At any time within the dialogue the facilitator or participants can propose a ‘time out’ in order to direct the attention of the group to any problems that may have arisen which prevents participants from focusing on the moral inquiry within the dialogue (Heckmann 1981; Loska 1995; Saran and Neisser 2004). For example, it may be that a participant is upset with the way the dialogue has developed; the group may have lost its way and need to review the structure or content of the dialogue. This is referred to as a ‘meta-dialogue’, which can be called for at any time (Boers 2003, p. 79). Within the meta-dialogue the actual situation in the group becomes the case for the moral inquiry (for as long as the meta-dialogue takes).

The idea of a time out or a meta-dialogue in the tradition of the Socratic dialogue is similar to the ‘disruption postulate’, in the methodology of TCI (Boers 2003). This disruption postulate implies that participants are invited to state when they are no longer able to continue the moral inquiry of the training or Socratic dialogue because of disruptions, tensions or intense emotions (Boers 2003, p. 84; see also Cohn 1976). Such an approach attempts to value differences, and conflicts between values among participants as opportunities to investigate these conflicts in the here-and-now. Such a time-out may be regarded as a moment which demands effort but an effort which is worthwhile (Wierdsma 1999, p. 130).

Trainers can use an intervention such as a meta-dialogue, or address a specific theme to work with, fitting to the situation. TCI can help to define a theme, in line with the development of a specific group. In the case described above, that could have been: ‘how can we deal constructively with other perspectives and conflicting values in this group?’. By addressing this theme, based on a TCI-analysis, all the participants are invited to share their experiences and their point of view on the issue that is at stake (Cohn 1976; Schneider-Landolf et al. 2009).

To overcome challenges and tensions of the learning process, such as a safety paradox, scholars have addressed the importance of creating a climate of support (Winnicot 1986). Creating a climate a climate of support may be fostered by paying attention to relevant structures or procedures. For instance, procedures indicate step by step how exactly participants will be working together to facilitate contributions from all participants. The mutual influence of structure on safety and process, in opposition to: chaos, unsafety and stagnation, is often stressed (Cohn 1976, p. 134). Specifically, for groups facing crisis phases, that may imply a risk of participants pulling out, supporting procedures may assist in reducing anxieties and feelings of unsafety (Cohn 1^976^; Schneider-Landolf et al. 2009).

While the trainers did sense that the critical incident described above was an important situation, they did not create time and space at that specific moment to discuss the situation in the here-and-now. Obviously, taking time and creating space immediately may not always be possible; neither will it always lead to the desired results. Nevertheless, we argue that it is important for trainers of ethics courses to learn to recognize these moments in which safety is at stake. Almost intuitively they will have to decide whether or not to create time and space to explicitly discuss a situations in which safety is at stake within the group. This can be described as the competence of a trainer to understand how different factors distinguished in the TCI approach, that is; individual characteristics, group dynamics, the course objective and the context are at play with regard to a specific situation. Moreover, the trainer should be able to make integrate experiences, emotions and thoughts in an intuitive hunch. Cohn refers to this competence as ‘trained intuition’ (Cohn 1976). This intuition may be strengthened when trainers regularly take time to reflect on safety dynamics in during training courses and analyze the situation in terms of the four factors.

## Conclusions

We argue that safety, understood as the feeling of a student that he or she can contribute to a dialogue or ask a critical question without fear or negative consequences, is an important precondition for ethics training courses. Safety is, however, precarious, and can be threatened. We showed that a safety paradox may occur during ethics training. This involves a tension between honesty and being critical on the one hand and openness to other perspectives and values on the other. Honesty may result in expressing conflicting or colliding views, which may challenge safety and mutual openness between participants. Approaching this paradox as a dynamic process requiring time and reflection in the here-and-now may assist to foster safety in those situations in which participants or trainers experience threats to safety. We argue that these situations offer an opportunity to learn during the training. Dealing constructively with other perspectives, conflicting values and remaining able to learn can be seen as key elements of ethics education. While it may not automatically lead to success, the four-factor analysis model can assist trainers in different contexts in both reflecting on these situations, as well as in preparing interventions and dealing with this paradox productively.
